# From Ivacaftor to Triple Combination: A Systematic Review of Efficacy and Safety of CFTR Modulators in People with Cystic Fibrosis

**DOI:** 10.3390/ijms21165882

**Published:** 2020-08-16

**Authors:** Andrea Gramegna, Martina Contarini, Stefano Aliberti, Rosaria Casciaro, Francesco Blasi, Carlo Castellani

**Affiliations:** 1 Department of Pathophysiology and Transplantation, University of Milano, 20129 Milano, Italy; stefano.aliberti@unimi.it (S.A.); francesco.blasi@unimi.it (F.B.); 2 Respiratory Disease and Adult Cystic Fibrosis Center, Internal Medicine Department, Fondazione IRCCS Ca’ Granda, Ospedale Maggiore, Policlinico, 20129 Milano, Italy; contarini.martina@gmail.com; 3 IRCCS Istituto Giannina Gaslini, Cystic Fibrosis Center, 16147 Genoa, Italy; rosariacasciaro@ospedale-gaslini.ge.it (R.C.); carlocastellani@gaslini.org (C.C.)

**Keywords:** *CFTR* modulators, clinical efficacy, safety, cystic fibrosis

## Abstract

Over the last years *CFTR* (cystic fibrosis transmembrane conductance regulator) modulators have shown the ability to improve relevant clinical outcomes in patients with cystic fibrosis (CF). This review aims at a systematic research of the current evidence on efficacy and tolerability of *CFTR* modulators for different genetic subsets of patients with CF. Two investigators independently performed the search on PubMed and included phase 2 and 3 clinical trials published in the study period 1 January 2005–31 January 2020. A final pool of 23 papers was included in the systematic review for a total of 4219 patients. For each paper data of interest were extracted and reported in table. In terms of lung function, patients who had the most beneficial effects from *CFTR* modulation were those patients with one gating mutation receiving IVA (ivacaftor) and patients with p.Phe508del mutation, both homozygous and heterozygous, receiving ELX/TEZ/IVA (elexacaftor/tezacaftor/ivacaftor) had the most relevant beneficial effects in term of lung function, pulmonary exacerbation decrease, and symptom improvement. *CFTR* modulators showed an overall favorable safety profile. Next steps should aim to systematize our comprehension of scientific data of efficacy and safety coming from real life observational studies.

## 1. Introduction

### 1.1. Cystic Fibrosis and the CFTR Protein

Cystic fibrosis (CF), the most common autosomal recessive disease in Caucasian populations, is a life-limiting condition, with respiratory failure secondary to end-stage lung disease accounting as the main cause of mortality. Early diagnosis through newborn screening, multi-professional care in dedicated centers, advancements in strategies of treatment, and availability of therapies have gradually improved median predicted survival up to 50 years [1].

CF is caused by mutations in the *Cystic Fibrosis Transmembrane Conductance Regulator* (*CFTR*) gene, first described together with its protein product in 1989 [2,3]. *CFTR* encodes a low conductance cAMP-dependent chloride channel located at the apical membrane of epithelial cells in several tissues including airways, the gastro-intestinal tract, sweat glands, and the male reproductive tract [4]. The protein includes two transmembrane domains (TMD1 and TMD2), two nucleotide-binding domains (NBD1 and NBD2), and a regulatory domain (RD). TMD1 and TMD2 run through the cell membrane lipid bi-layer and combine to form the channel; each unit is formed by six distinct segments and three extracellular loops, that undergo a coordinated folding process to reach the correct three-dimensional stability. The RD presents sites for phosphorylation by both protein kinase C (PKC) and A (PKA). Once the RD is phosphorylated, two ATP molecules bind to the NBD1 and NBD2 heterodimers and the channel opens, enabling anion conductance through the pore [5]. More than 2000 *CFTR* sequence variations have been identified, but in only a few hundred a clear association with loss of function and clinical manifestations consistent with a diagnosis of CF has been demonstrated. These variants are called CF-causing mutations (www.cftr2.org).

### 1.2. A Classification for CFTR Mutations

*CFTR* mutations are usually grouped according to their molecular mechanisms of dysfunction [6,7].

Class I includes mutations affecting protein synthesis, mostly represented by nonsense mutations resulting in a premature stop codon and in mRNA decay.

Class II mutations operate mainly on trafficking and include p.Phe508del, the most prevalent pathogenic sequence variation in Western countries, that originates from the loss of a single phenylalanine at the 508th position in the protein sequence. The protein is incorrectly folded and rapidly degraded, which impairs protein trafficking to the cell surface and results in severe reduction of *CFTR* activity.

Class III mutations, the so-called gating mutations, diminish the open probability of the channel: the gate-open probability of p.Gly551Asp, the most prevalent gating mutation, is more than 100-fold lower than wild-type.

Class IV mutations, of which a frequent example is p.Arg117His, reduce chloride and bicarbonate flow through the channel, but still permit a degree of residual function.

Class V mutations decrease amount and activity of *CFTR* at the cell surface, mainly through alternative splicing that generates both normal and aberrant mRNA and functional protein. The proportion of normal and aberrant protein might vary among patients and among different organs in the same patient.

Class VI mutations affect *CFTR* stability at the plasma membrane level, thus reducing the protein expression and recycling at the apical surface. 

Sequence variations that result in limited but not absent *CFTR* function, that is classes IV-VI, are called ‘residual function’ mutations. 

Frequently, mutations feature more than one mechanism of protein failure and thus belong to more than one class. A typical example is p.Phe508del, which results not only in trafficking defect (class II) but also in reduced activity (class III) and stability at the plasma membrane level (class VI). Although this functional classification has proven only partially useful to predict individual clinical outcomes, it has been successfully used for new approaches in translational research.

### 1.3. Drug Development

*CFTR* function may be partially rescued by molecules known as modulators, which include potentiators (ivacaftor), that increase conductance of the *CFTR* channel, and correctors (lumacaftor, tezacaftor, elexacaftor), that improve *CFTR* trafficking to the cell surface.

Ivacaftor (IVA) was the first of these molecules that proved effective in a phase III clinical trial, with significant pulmonary and nutritional improvements, and paved the way to a new generation of precision medicine drugs in CF [8]. In 2015 the North American and European regulatory agencies licensed a combination of ivacaftor and lumacaftor (LUM), the latter being a corrector of p.Phe508del folding and trafficking defect. In spite of a modest improvement of lung function and other clinical outcomes, this association had the merit to target for the first time the most common *CFTR* mutation and widen the population treatable with modulators [9].

Following the increased knowledge on the functioning of *CFTR* modulators and the development of alternative experimental models, new compounds have been experimented. For patients with p.Phe508del and a residual function mutation in *trans*, treatment with tezacaftor/ivacaftor (TEZ/IVA) was effective in terms of lung function and resulted in a significantly lower rate of pulmonary exacerbations than placebo [10].

However, neither of these double combinations was found to be satisfactorily effective in patients carrying a single p.Phe508del and a second *CFTR* mutation that does not respond to previous *CFTR* modulator therapy and so-called ‘minimal function’ mutations. VX-659 and VX-445 (elexacaftor, ELX) are next-generation correctors with different mechanisms of action than previous generation correctors LUM and TEZ. 

Co-treatment with two complementary correctors proved to be the most effective strategy to improve the expression of corrected p.Phe508del *CFTR* protein at the cell surface on the basis of both in vitro activity and clinical results [11,12]. ELX and TEZ, along with IVA, has recently extended the treated population also to those with a *CFTR* minimal function mutation [13]. In addition, both TEZ/IVA and ELX/TEZ/IVA showed better results than LUM/IVA in p.Phe508del homozygous patients [14,15].

This review aims at a systematic research of the current evidence on efficacy and tolerability of *CFTR* modulators nowadays available for different genetic subsets of patients with CF.

## 2. Methods

### 2.1. Search Methodology

This systematic revision was conducted according to the PRISMA statement [16]. Two investigators (AG and MC) independently performed the research on PubMed and screened the literature in order to identify phase 2 and 3 studies published in the study period 1 January 2005–31 January 2020. Key phrases included ‘cystic fibrosis’ or ‘*CFTR*’ and ‘clinical trial’; ‘cystic fibrosis AND modulators’. The research was extended also to other databases (EMBASE, Cochrane Central Register for Controlled Trials, Cochrane Database of Systematic Reviews). In order to increase the search sensitivity, the reference lists of the selected papers were assessed also manually. Non peer-reviewed papers were not selected due to poor methodological reliability.

### 2.2. Study Selection

After literature search, two independent investigators reviewed titles and abstracts in order to select those that fulfilled the study criteria; in case of disparity, a final decision was taken by a third reviewer (SA). The authors included only phase 2 and 3 clinical trials published in the mentioned study period. In accordance with the inclusion criteria mentioned above, literature on new modulators dealing with in vitro and preclinical data as well as phase 1 clinical trials, although of interest, was excluded. Phase 2 trials that have been completed with results but not yet undergone the peer-review process and publication were excluded from the systematic analysis. Articles were also excluded if (1) written in languages other than English; (2) they were abstracts presented in national and international congresses; (3) they were commentaries, correspondences, editorials, case-series. The full text was obtained for selected papers.

### 2.3. Data Extraction

Data of interest were extracted from each included paper. Qualitative and quantitative data were extracted by the same reviewers (AG and MC) who performed the study selection, with the help of a third reviewer (SA) if needed. Data of interest included name of the first author, year of publication, phase of study, study population and sample size, type of intervention, duration, primary and secondary endpoints, report of adverse events (AEs), and number of patients who interrupted the study drug due to the occurrence of any AE. Corresponding authors were contacted if data were unclear or not reported in the full text. In consideration of the heterogeneity of the papers, a meta-analysis was not performed.

## 3. Results

### 3.1. Study Selection and Characteristics

**Figure 1:**
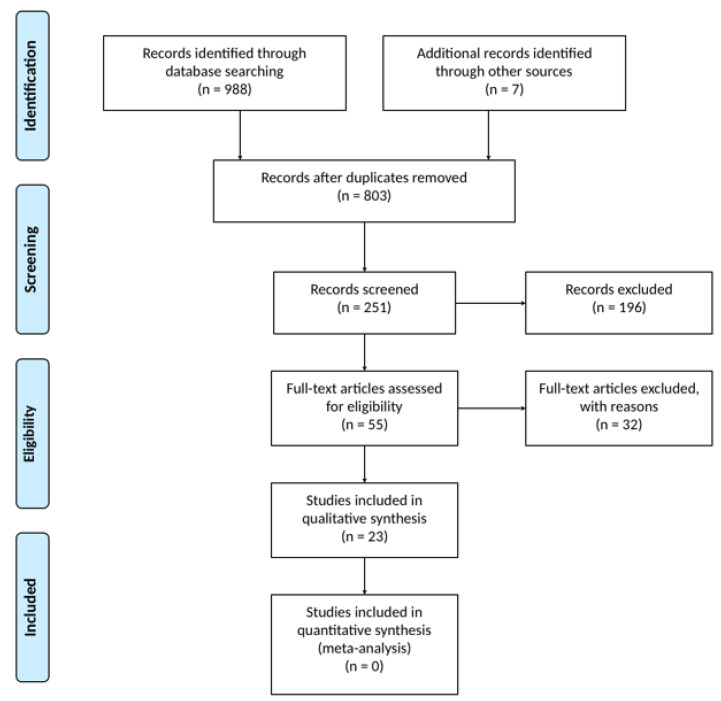
Flow chart of the systematic review.

Figure 1 shows the selection process and the search results. Qualitative reviews, retrospective analysis, and in vitro experiments were rejected. A pool of 23 studies was included in the systematic review with a total of 4219 (age 6–11: 436; age 12+: 3783) patients. The papers selected were published between the years 2005 and 2020. We included 11 phase 2 and 12 phase 3 clinical trials. Characteristics of selected clinical trials are reported in Table 1.

### 3.2. Primary and Secondary Outcomes

Analysis of outcome measures is reported in Table 1. Most studies assessed as primary outcome the improvement in lung function, measured by the absolute change in ppFEV1 from baseline through the study period (*n* = 13, 68%). A second group of studies, mainly phase 2 clinical trials, evaluated safety and tolerability as their primary outcome (*n* = 7, 37%). Sweat chloride concentration and number of pulmonary exacerbations (PEX) were the most prevalent secondary outcomes in 15 (79%) and six (31%) studies, respectively. Patient-reported outcomes (PROs) were considered as primary or secondary outcomes in 19 studies (83%). In all cases PROs were evaluated by the use of CFQ-R Respiratory domain score.

### 3.3. Lung Function

Patients who had the most beneficial effects from *CFTR* modulators were those with one gating mutation receiving IVA and those with p.Phe508del mutation, both homozygous and heterozygous, receiving ELX/TEZ/IVA [8,13,15].

IVA 150 mg BID was associated with increase in ppFEV1 compared to placebo in patients carrying p.Gly551Asp mutation, both aged 6–11 years (ppFEV1 +12.5 points) and ≥12 years (ppFEV1 +10.6 points) [8,20]. IVA increased lung function compared to placebo (ppFEV1 +8.3 points) in patients aged ≥6 years carrying non-p.Gly551Asp gating mutations [23]. IVA was also reported to improve ppFEV1 (+2.1 points) in patients ≥6 years with R117H [24].

Treatments with IVA alone and LUM alone were not effective on lung function in p.Phe508del homozygous patients in two different phase 2 clinical trials, while the LUM/IVA association increased ppFEV1 in both phase 2 and phase 3 trials in patients homozygous for p.Phe508del (+6.1 and +4.8 points, respectively) [9,18,19,22].

TEZ/IVA significantly improved FEV1 in patients ≥12 years both p.Phe508del homozygous and p.Phe508del along with a residual function mutation in comparison to placebo (ppFEV1 +4.0 and +6.8 points, respectively) [10,14]. In this last study group, TEZ/IVA was associated with ppFEV1 improvement of 2.1 points compared to IVA alone [10]. No change in FEV1 was described for TEZ alone in both populations [27].

Two triple combinations of either VX-659 or VX-445 (ELX) plus TEZ/IVA resulted in an overall improvement in ppFEV1 compared to placebo in two phase 2 clinical trials recruiting patients ≥12 years both p.Phe508del homozygous (9.7 and 11.0 points, respectively) and p.Phe508del along with a minimal function mutation (13.3 and 13.8 points, respectively) [11,12]. Treatment with ELX/TEZ/IVA confirmed significant improvements of lung function in the same patient populations with ppFEV1 increase over baseline of 10.4 and 13.8, respectively [13,15].

Referring to other genetic subsets, ataluren did not lead to any significant change in ppFEV1 in a population of patients aged 6 years or more carrying at least one non-sense mutation [21].

Change in Lung Clearance Index (LCI) was also considered as primary outcome by one single clinical trial [26]. Treatment with LUM/IVA demonstrated a slight but significant improvement in lung ventilation measured as change of LCI 2.5 from baseline (−1.09 units) compared to placebo in patients homozygous for p.Phe508del and aged 6–11 years.

### 3.4. Pulmonary Exacerbation

IVA demonstrated a 55% reduction in the risk of pulmonary exacerbation compared to placebo in patients ≥12 years receiving p.Gly551Asp [8]. The combination of LUM/IVA in patients homozygous for p.Phe508del decreased both PEX and PEX conditioning hospitalization of 39% and 61%, respectively [9]. Lower annualized rate of pulmonary exacerbations in comparison to placebo was also demonstrated for p.Phe508del homozygotes receiving TEZ/IVA (−35%) and ELX/TEZ/IVA (−63%) [14,15].

On the contrary, ataluren did not result in significant reduction of PEX episodes compared to placebo for patients with at least one non-sense mutation in a phase 3 clinical trial [21].

### 3.5. Sweat Chloride

Sweat chloride concentration was a relatively common secondary outcome in trials evaluating *CFTR* modulators. The greatest improvement in sweat chloride was described in patients carrying one gating mutation receiving IVA (48.1 mmol/L in p.Gly551Asp and 49.2 mmol/L in other gating mutations) and those with p.Phe508del mutation, both homozygous and heterozygous, receiving ELX/TEZ/IVA (43.4 mmol/L in homozygotes and 41.2 mmol/L in heterozygotes) [8,13,15,23].

Significant decreases in sweat chloride concentrations were described for p.Phe508del homozygous patients with both the corrector ABBV-2222 (formerly GLPG2222) alone and the potentiator GLPG2737 on top of LUM/IVA (−15.8 and −11.7 mmol/L, respectively) [29,31]; and for p.Gly551Asp patients with the potentiator GLPG1837 after IVA wash-out (−28.8 mmol/L) [30].

### 3.6. Patient-Reported Outcomes

Patients homozygous for p.Phe508del and patients with p.Phe508del and a minimal function mutation in *trans* receiving ELX/TEZ/IVA had the most significant improvements on quality of life in terms of CFQ-R Respiratory domain improvement (20.7 and 25.7 points compared to placebo, respectively) [13,15].

Compared to placebo, IVA improved quality of life with similar magnitude in three different groups: patients with p.Gly551Asp, patients with gating mutations other-than-p.Gly551Asp, and patients with p.Arg117His mutation (8.6, 9.6, and 8.4 points in CFQ-R, respectively) [8,23,24]. Treatment with TEZ/IVA was associated with CFQ-R increase of 11.1 points in patients with a residual function mutation and 5.1 points in p.Phe508del homozygotes [10,14]. Significant but lower improvements in CFQ-R were reported for patients receiving LUM/IVA (2.2 points) [9]. There was no improvement in CFQ-R Respiratory domain score in comparison to placebo for p.Phe508del homozygous patients on IVA and for patients aged 6–11 years on LUM/IVA [18,26]. Furthermore, ABBV-2222 on top of IVA in patients with one gating mutation and GLPG2737 on top of LUM/IVA in patients homozygous for p.Phe508del did not lead to increase in CFQR-R compared to placebo [29,31].

### 3.7. Safety

The highest rate of study drug discontinuation was reported during treatment with ataluren in patients carrying a non-sense mutation (3.4%) [21]. Patients homozygous for p.Phe508del and treated with LUM/IVA experienced drug discontinuation in 2.8% of study cohort resulting as the highest discontinuation rate among *CFTR* modulators approved for clinical use [9]. The most frequent adverse events were respiratory events including PEX, increase in cough or expectoration, upper respiratory tract infections, or hemoptysis.

Regarding extra-respiratory events, headache and diarrhea were the most reported.

Table 2 summarizes the 15 most common adverse events across studies involving the four CFT modulators currently available in clinical practice.

## 4. Discussion

To the best of our knowledge, this is the most inclusive systematic review on efficacy and safety of *CFTR* modulators in people with CF, including also data from recent clinical trials on the triple combination therapy [32].

### 4.1. Expansion of the Target Population

Clinical studies on *CFTR* modulators span over a period of about 10 years, with a gradual enlargement of the genotypes reached by the trials. While in the 2010–2013 period several studies focused on IVA in patients carrying p.Gly551Asp, later the most tested compounds were LUM/IVA and TEZ/IVA in individuals with p.Phe508del/p.Phe508del or p.Phe508del/any genotypes, that together account for up to 85% of the affected alleles in North America and Europe [33].

A further expansion of the therapeutic indications of these compounds took place along three main research paths. First, testing the highly effective IVA in patients carrying mutations sharing the same class 3 functional classification as p.Gly551Asp or preserving some residual function. That was the case for IVA, that was demonstrated effective in patients carrying non-Gly551Asp gating mutations or the p.Arg117His mutation [23,24]. In 2017, FDA approved the extension of the use of IVA to treat additional *CFTR* mutations based on results from an in vitro cell-based model system [34].

Second, *CFTR* modulators previously tested for safety and efficacy in adults were examined in a subset of the pediatric population, as in the case of Davies and Ratjen, who tested IVA and LUM/IVA in 6–11 years old children [20,26]. Only eight studies out of a total of 19 exclusively enrolled adults (age 18+), while the majority involved a mixed population of adolescents, younger adults, and adults with CF (age 12+). Overall, a total of 11 studies, that is a slight majority, involved groups of patients with age <18 years old.

Finally, randomized controlled trials (RCTs) have been carried out to examine new molecules and combinations [10,11,12,14]. Following the controversial clinical effects of lumacaftor (LUM), additional research through high-throughput screening has been performed to identify next-generation correctors [35]. To date, TEZ and ELX demonstrated the best pharmacological properties and clinical efficacy in rescuing p.Phe508del *CFTR*. ELX in particular allowed to target new CF subpopulations including those carrying p.Phe508del and either residual or minimal function mutations.

### 4.2. Efficacy on Lung Function

Before the triple combination became available, of all CF genotypes it was IVA in patients with p.Gly551Asp who reached the best primary endpoint results in a clinical trial [8]. Long-term treatment with IVA in patients with gating mutations has been also associated with significant decrease in mortality and in need for lung transplantation [36].

The degree of lung function and PEX improvement in p.Gly551Asp patients receiving IVA has been for a few years the unapproachable benchmark and considered the only highly effective *CFTR* modulator [8]. Recently, the triple combination in p.Phe508del homozygotes and in p.Phe508del heterozygotes whose second mutation has minimal function showed clinical benefits of a magnitude similar to those of p.Gly551Asp treated with IVA. In fact, the latter group of patients in the phase 3 clinical trial achieved a 10.6 increase in ppFEV1 compared to placebo, and patients with two or one copy of p.Phe508del treated with ELX/TEZ/IVA experienced similar gains in ppFEV1, of 10.0 and 14.3, respectively [8,13,15]. These results acquire a particularly valuable clinical meaning considering that before triple combination patients homozygous for p.Phe508del and treated with LUM/IVA (6+ years old) or TEZ/IVA (12+ years old) had much smaller increases in lung function (2.8 and 4 percentage points, respectively) in comparison to placebo [9,14].

In relation to residual function genotypes, the phase 3 study EXPAND showed an increase in ppFEV1 of 6.8 percentage points for patients with p.Phe508del in *trans* with a residual function mutation and treated with TEZ/IVA [10]. The ongoing study of ELX in association with TEZ and IVA in subjects with p.Phe508del and a gating or residual function mutation will confirm whether or not triple combination could be extended even to patients already treated with an effective *CFTR* modulator therapy (https://clinicaltrials.gov/, NCT04058353).

Although ivacaftor has demonstrated efficacy in achieving relevant clinical endpoints, it should also be noted that an effort to develop better *CFTR* potentiators is underway. These potential new treatments have shown promising results in clinical trials [29,30,31].

### 4.3. Efficacy on PEXs

The efficacy of *CFTR* modulators in PEX reduction was most relevant for p.Gly551Asp patients treated with IVA (−55% PEX frequency) and p.Phe508del/minimal function patients treated with triple combination (−63% PEX frequency) [8,15]. Although PEXs were not an efficacy outcome in the 4-week trial of triple combination in p.Phe508del homozygotes, there was a decrease in respiratory-related events in ELX/TEZ/IVA group compared with TEZ/IVA group [13,14]. Open-label extension studies will explore the reproducibility of this outcome in patients with p.Phe508del homozygosity and over a longer period of time.

### 4.4. Efficacy on Patient Reported Outcomes (PROs)

The magnitude of improvement in quality of life, and especially in the respiratory symptoms domain, was in line with the increase in ppFEV1 across studies, thus confirming from a patient perspective the clinical results.

### 4.5. Safety

Respiratory-related events were reported most frequently for LUM/IVA, which was also the only compound associated with chest tightness. In 2017, Popowicz demonstrated that ppFEV1 acutely dropped of a mean of 19 percentage points (range −21% to –11%, *p* = 0.001) at 2 h after LUM/IVA initiation in patients with p.Phe508del homozygosity and moderate to severe lung function impairment [37]. These events are consistent with the relatively high number of patients who discontinued LUM/IVA during RCTs or open label extended studies [9,38]. Some authors interpreted chest tightness and bronchospasm as a marker of therapeutic efficacy, as mucus fluidification and swelling following LUM/IVA administration might cause airway obstruction until phlegm is eventually expelled. However, a direct bronchospastic effect might be a more realistic explanation, in the light that TEZ/IVA and the new triple combination therapy are better tolerated than LUM/IVA both in RCTs and real life.

Abnormal liver function tests have been reported as common adverse events in patients treated with *CFTR* modulators, both in children and adult populations; most of the events were low-to-moderate in severity and did not require drug discontinuation.

ELX/TEZ/IVA combination therapy was also associated with skin rash, both in the p.Phe508del homozygous and heterozygous study [13,15]. In the latter trial, the rash occurred in 22 patients (10.9%) in the triple combination group and 13 patients (6.5%) in the placebo group. All events were defined as low grade adverse events and resolved during the trial. Notably, in both trial groups rash was more common in women receiving a concomitant hormonal oral contraceptive.

## 5. Unanswered Questions

The introduction of *CFTR* modulators in clinical practice has produced a significant impact on short-term clinical outcomes in people with cystic fibrosis. However, high internal validity and strict inclusion criteria in RCTs inevitably lead to low representativeness of everyday clinical practice.

An open question is if there are substantial effects in the severe patient subgroup that did not meet lung function inclusion criteria and could not participate in the trials. Encouraging data come from a recent cumulative analysis of outcomes from clinical trials, taking into consideration patients whose ppFEV1 declined below 40 between screening and the randomization visit, and open-label extension studies [39]. However, severity was merely defined as ppFEV1 <40 and did not fully represent the wide range of real-life circumstances, like patients awaiting lung transplantation, continuous IV antibiotic therapy, recurrent life-threatening hemoptysis, or NTM pulmonary disease.

On the other side, no data are available about the effects of *CFTR* modulators on people with very mild or no pulmonary involvement. More evidence on this topic would be useful to clinicians in order to guide the choice on the best timing to initiate *CFTR* modulators in the absence of respiratory disease.

Since ELX/TEZ/IVA proved to be a new highly effective treatment for people with p.Phe508del mutation, many issues have been rising including its possible extension to treat rare mutations that have currently no indications to *CFTR* modulators. Both translational experiments as well as further understanding of the molecular basis of *CFTR* misfolding are now encouraged to answer this question.

A further question that lies unresolved is for how long clinical benefits secondary to *CFTR* modulation can be maintained. A global evaluation of open-label extended studies and real life reports from national registries might assess the long-term sustainability of the clinical benefits demonstrated in RCTs and the overall impact not only on lung health but also extra-respiratory involvement, co-morbidities, and mortality. Next steps should aim to systematize our comprehension of scientific data of efficacy and safety coming from real life observational studies.

## 6. New Strategies beyond *CFTR* Potentiators and Correctors

The molecules included in this systematic review all belong to the two categories of potentiators and correctors of *CFTR* protein along with their combinations. However, novel approaches to post-translational modifications of *CFTR* protein are being explored in order to both address orphan mutated alleles and optimize correction of p.Phe508del mutant protein. A complementary therapeutic strategy is based upon the fact that the effectiveness of correctors and potentiators not only depends on their potential to rescue *CFTR* function, but also on the quantity of protein to operate on. The assumption is that augmenting the pool of *CFTR* available for modulation might translate into greater function improvements. A recent high-throughput screening identified a new class of modulators acting neither as potentiators nor correctors and termed amplifiers. These compounds have *CFTR* shown to increase the expression of *CFTR* mRNA and immature *CFTR* protein across different *CFTR* classes and mutations, including p.Phe508del [40]. Nesolicaftor (PTI-428, Proteostasis) is the first amplifier tested both in vitro and in clinical trials. A study on HBE cell lines and patient-derived nasal cultures from Molinski and al. demonstrated a significantly higher increase in *CFTR* mRNA levels when the combination of PTI-428 and LUM was used in comparison to LUM treatment alone [41]. A phase 2 trial of nesolicaftor in combination with TEZ/IVA therapy in individuals with CF and homozygous for the p.Phe508del mutation has been recently completed. Although PTI-428 did not reach the expected lung function endpoint, the authors demonstrated an increase of approximately 50% in *CFTR* production (NCT03591094) [42]. Nesolicaftor has also been studied in triple combination with two other *CFTR* correctors, PTI-801 and PTI-808 (Proteostasis Therapeutics). The phase 2 study in patients with two copies of the p.Phe508del mutation showed a significant 5 points increase in ppFEV1 (NCT03500263). Proteostasis announced it will start two phase 3 clinical trials to follow up with the promising success of this triple combination, in p.Phe508del homozygotes.

Another therapeutic approach aims at stabilizing the mutant *CFTR* protein which, after proper trafficking to the cell surface, is already included in the membrane. The targets are variants with reduced stability, like Class VI mutations or LUM-rescued p.Phe508del protein, where the misfolding of the mutant protein prevented recycling and promoted lysosomal targeting and accelerated endocytosis [43]. A novel class of *CFTR* modulators, named stabilizers, exerts its effect by targeting conformation-dependent ubiquitines and other molecules involved in the peripheral quality control, thus increasing half-life of the *CFTR* channel and preventing premature degradation [44]. Several different molecules have demonstrated to improve *CFTR* stabilization, including the hepatocyte growth factor (HGF), the vasoactive intestine peptide (VIP), and the inhibitors of S-nitrosoglutathione reductase (GSNOR). Among them, cavosonstat (N91115, Nivalis Therapeutics) showed strong inhibitory activity against GSNOR, thus preserving levels of *S*-nitrosoglutathione and positively affecting the stability of *CFTR* [45]. The same compound together with *CFTR* correctors showed in vitro a significant increase in the expression and activity of p.Phe508del [46]. In a recent phase 1 trial cavosonstat was well tolerated and demonstrated significant reduction in sweat chloride when administered at the highest tested dose [47]. However, in a series of two phase 2 trials cavosonstat combined with IVA and LUM/IVA did not demonstrate significant effects on lung function (NCT02724527 and NCT02589236, respectively). To the best of our knowledge, no further clinical development for cavosonstat is planned at this time.

## Figures and Tables

**Table 1:** Results of the systematic review.

Author, Year	Phase	Study Population	Arms	Duration	Results (1 = Primary Outcome; 2 = Key Secondary Outcomes)	Patients Who Interrupted the Study Drug Due to AEs
Accurso, 2010 [17]	2	*CFTR*: at least one G551D Age: 18+ Sample size: 20 (first part) and 19 (second part)	*First part* IVA 25 mg BID IVA 75 mg BID IVA 150 mg BID Placebo *Second part* IVA 150 mg BID IVA 250 mg BID Placebo	*First part* Two 14 day periods *Second part* 28 days	(1) The frequency of adverse events was similar between the groups *At day 28 in IVA 150 mg group:* (2) NPD: −3.5 mV (−8.3, −0.5) (2) ST: −59.2 mmol/L (−66, −19) (2) FEV1: 8.7% (2.3, 31.3) (2) CFQ-R: 8.3 (0, 16.7)	No discontinuations due to AEs
Ramsey, 2011 [8]	3	*CFTR*: at least one G551D Age: 12+ Sample size: 161	IVA 150 mg BID Placebo	48 weeks	(1) absolute change in ppFEV1 (week 24): 10.6% (2) PEx decrease (week 48): −55% (2) CFQ-R: +8.6 points (2) BMI: +2.7 kg (2) ST: −48.1 mmol/L	One patient discontinued the study drug for increased LFTs (0.6%)
Flume, 2012 [18]	2	*CFTR*: F/F Age: 12+ Sample size: 140	IVA 150 mg BID Placebo	16 weeks	(1) absolute change in ppFEV1: 1.7% (−0.6, 4.1) (2) ST: -2.9 (-5.6, -0.2) (2) BMI: no significant change (2) CFQ-R: no significant change	No discontinuations due to AEs
Clancy, 2012 [19]	2	*CFTR*: F/F Age: 18+ Sample size: 89	LUM 25 mg SID LUM 50 mg SID LUM 100 mg SID LUM 200 mg SID Placebo	28 days	(1) safety: no difference in AEs between groups (2) ST: −6.6 (−10.27, −2.83) (2) NPD: no significant change	Four patients discontinued the study drug (respiratory AEs)
Davies, 2013 [20]	3	*CFTR*: at least one G551D Age: 6–11 Sample size: 52	IVA 150 mg BID Placebo	48 weeks	(1) absolute change in ppFEV1 through week 24: 12.5 (6.6, 18.3) (2) absolute change in ppFEV1 through week 48: 10 (4.5, 15.5) (2) weight at weeks 24 and 48: 1.9 (0.9, 2.9) and 2.8 (1.3, 4.2) (2) ST through weeks 24 and 48: −54.3 (−61.8, −46.8); −53.5 (−60.9, -46) (2) CFQ-R through weeks 24 and 48: 6.1 (−1.4, 13.5); 5.1 (−1.6, 11.8) (2) safety: no difference in AEs between groups	One patient discontinued the study drug (1.9%)
Kerem, 2014 [21]	3	*CFTR*: at least one NS Age: 6+ Sample size: 238	Ataluren TID Placebo	48 weeks	(1) no significant change in ppFEV1 (2) no significant reduction in PEx rate (2) no significant change in ST and NPD	Eight patients discontinued the study drug (3.4%)
Boyle, 2014 [22]	2	*Cohort 1*: *CFTR*: F/F Age: 18+ Sample size: 62 *Cohort 2*: *CFTR*: F/F Age: 18+ Sample size: 82 *CFTR*: F/any mutation Age: 18+ Sample size: 27 *Cohort 3*: *CFTR*: F/F Age: 18+ Sample size: 15	*Cohort 1:* LUM 200 mg SID/IVA 150 mg BID LUM 200 mg/IVA 250 mg BID Placebo *Cohort 2, F/F*: LUM 200 mg SID/IVA 250 mg BID LUM 400 mg SID/IVA 250 mg BID LUM 600 mg SID/IVA 250 mg BID Placebo *Cohort 2, F/any mutation*: LUM 600 mg/IVA 250 mg Placebo *Cohort 3*: LUM 400 mg BID/IVA 250 mg BID	14 days of LUM followed by 7 days of LUM/IVA or Placebo 21 days 28 days of LUM followed by 28 days of LUM/IVA Placebo for 56 days 28 days of LUM followed by 28 days of LUM/IVA Placebo for 28 days 28 days of LUM followed by 28 days of LUM/IVA Placebo for 56 days	*Cohort 1*: (1) ST: −12.6 (−17.2, −7.9) for IVA 250 mg vs. day 1 −10.9 (−17.6, −4.2) for IVA 250 mg vs. placebo (1) Safety: no difference in AEs between groups (2) absolute change in ppFEV1 at days 7, 14, and 21: not significant change in LUM, LUM/IVA 250; 3.1 (0.1–6.1) in LUM/IVA 150 at day 21 (2) ST at day 14: data not presented (2) PK: data not presented *Cohort 2:* (1) ST: −9.1 (−13.3, −4.9) in LUM 400 SID −8.9 (−13.1, −4.7) in LUM 600 (1) Safety: no difference in AEs between groups (2) absolute change in ppFEV1 at days 14, 28, 42, and 56: not significant change in LUM; 6.2 (3.3–9) in LUM 600/IVA 2) ST at days 28 and 56: data not presented 2) CFQ-R at days 14, 28, 42, and 56: 15.9 (5.8, 26) in LUM 200; 13.5 (3.2, 23.9) in LUM 400 (2) PK: data not presented *Cohort 3*: (1) ST: −10.3 (−16.7, −4) (1) Safety: no difference in AEs between groups (2) absolute change in ppFEV1 at days 14, 28, 42, and 56: 6.1 (2, 10.2) in LUM 400 BID/IVA 259 BID (2) ST at days 28 and 56: data not presented (2) CFQ-R at days 14, 28, 42, and 56: data not presented (2) PK: data not presented	No discontinuations due to AEs
De Boeck, 2014 [23]	3	*CFTR*: at least one non-G551D gating mutation Age: 6+ Sample size: 39	IVA 150 mg BID Placebo	8 weeks	(1) absolute change in ppFEV1: 8.3 (4.5, 12.1) (2) BMI: 0.7 kg/m2 (0.34, 0.99) (2) ST: −49.2 (−57, −41.4) (2) CFQ-R: 9.6 (4.5,14.7)	No discontinuations due to AEs
Wainwrigth, 2015 [9]	3	*CFTR*: F/F Age: 12+ Sample size: 1108 (549 in the TRAFFIC study and 559 in the TRANSPORT study)	LUM 600 mg SID/IVA 250 mg BID LUM 400 mg BID/IVA 250 mg BID Placebo	24 weeks	(1) absolute change in ppFEV1: 2.8 (1.8, 3.8) (2) relative change in ppFEV1: 4.8 (3.0, 6.6) (2) BMI: 0.24 (0.11, 0.37) (2) CFQ-R: 2.2 (0.0, 4.5) (2) PEx: 0.39 (0.49, 0.76	In total, 31 patients discontinued the study drug for elevation of the creatine kinase level in four patients, hemoptysis in three patients, bronchospasm in two patients, dyspnea in two patients, pulmonary exacerbation in two patients, and rash in two patients (2.8%)
Moss, 2015 [24]	3	*CFTR*: at least one R117H Age: 6+ Sample size: 69	IVA 150 mg BID Placebo	24 weeks	(1) absolute change ppFEV1: 2.1 (1.13, 5.35) 2) ST: −24.0 mmol/L (−28.0, −19.9) (2) CFQ-R: 8.4 (2.2–14.6)	No discontinuations due to AEs
Rowe, 2017 [25]	2	*CFTR*: F/ unresponsive to ivacaftor CFTR mutation Age: 18+ Sample size: 126	LUM 400 mg BID/IVA 150 mg BID Placebo	56 days	(1) absolute change in ppFEV1: no significant change (2) no significant change in CFQ-R (2) no change in BMI (2) no change in FEV1	Four patients discontinued the study drug for respiratory related AEs (3.2%)
Taylor-Cousar, 2017 [14]	3	*CFTR*: F/F Age: 12+ Sample size: 504	TEZ/IVA 100 mg SID/150 mg BID Placebo	24 weeks	(1) absolute change in ppFEV1: 4.0% (3.1, 4.8) (2) relative change in ppFEV1: 6.8% (5.3, 8.3) (2) CFQ-R: 5.1 points (3.2–7.0) (2) decrease in PEx: −35% (2) no difference in BMI	No discontinuations due to AEs
Ratjen, 2017 [26]	3	*CFTR*: F/F Age: 6–11 years Sample size: 206	LUM 200 mg BID/IVA 250 mg BID Placebo	24 weeks	(1) absolute change in LCI2.5 from baseline: −1.01 (−1∙27, −0.75) (2) ST: −20.8 (−23.4, −18.2) (2) BMI: 0.38 (0.25, 0.52) (2) CFQ-R: not significant change (2) ppFEV1: not significant change	Two patients discontinued the study drug for elevated liver enzymes in one patient, rash in one patient (1%)
Rowe, 2017 [10]	3	*CFTR*: F/RF Age: 12+ Sample size: 246	TEZ 100 mg SID/IVA 150 mg BID IVA 150 mg BID Placebo	Two treatment periods of 8 weeks separated by a washout period of 8 weeks	(1) absolute change in ppFEV1 at average of weeks 4 and 8: TEZ/IVA vs. placebo: 6.8 (5.7, 7.8) IVA vs. placebo: 4.7 (3.7, 5.8) TEZ/IVA vs. IVA: 2.1 (1.2, 2.9) (2) absolute change in CFQ-R at average of weeks 4 and 8: TEZ/IVA vs. placebo: 11.1 (8.7, 13.6) IVA vs. placebo: 9.7 (7.2, 12.2) TEZ/IVA vs. IVA: 1.4 (−1, 3.9) (2) safety and tolerability: (2) relative change in ppFEV1 at average of weeks 4 and 8: TEZ/IVA vs. placebo: 11.4 (9.6, 13.2) IVA vs. placebo: 8.1 (6.3, 9.9) TEZ/IVA vs. IVA: 3.3 (1.8, 4.8) (2) absolute change in ST at average of weeks 4 and 8: TEZ/IVA vs. placebo: −9.5 (−11.7, −7.3) IVA vs. placebo: −4.5 (−6.7, −2.3) TEZ/IVA vs. IVA: −5.1 (−7, −3.1)	No discontinuations due to AEs in the study group
Davies, 2018 [11]	2	*CFTR*: F/MF Age: 18+ Sample size: 88 *CFTR*: F/F Age: 18+ Sample size: 29	*F/MF:* VX-659 80 mg SID/TEZ 100 mg SID/IVA 150 mg BID VX-659 240 mg SID/TEZ 100 mg SID/IVA 150 mg BID VX-659 400 mg SID/TEZ 100 mg SID/IVA 150 mg BID Placebo VX-659/TEZ/VX-561* SID Placebo *F/F*: VX-659 400 mg SID/TEZ 100 mg/IVA 150 mg BID TEZ 100 mg SID/IVA 150 mg BID	4 weeks followed by 4 days of TEZ/IVA 4 weeks 4 weeks followed by 4 weeks of TEZ/IVA	(1) safety and side-effects: not significant differences (1) absolute change in ppFEV1 through day 29: VX-659 80 mg: 10.2 (4.8, 15.5) VX-659 240 mg: 12 (8, 16) VX-659 400 mg: 13.3 (9.5, 17.1) (2) absolute change in ST through day 29: VX-659 80 mg: −45.7 (−54.4, −37) VX-659 240 mg: −43.8 (−50.7, −37) VX-659 400 mg: −51.4 (−57.8, −44.9) (2) absolute change in CFQ-R at day 29: VX-659 80 mg: 24.6 (13, 36.2) VX-659 240 mg: 19.8 (11, 28.6) VX-659 400 mg: 21.8 (13.6, 30) (1) safety and side-effects: not significant differences (1) absolute change in ppFEV1 through day 29: 12.2 (8.3, 16.2) (2) absolute change in ST through day 29: −38.1 (−44.4, −31.8) (2) absolute change in CFQ-R at day 29: 14.7 (7.1, 22.4) (1) safety and side-effects: not significant differences (1) absolute change in ppFEV1 through day 29: 9.7 (6.6, 12.7) (2) absolute change in ST through day 29: −42.2 (−46.8, −37.7) (2) absolute change in CFQ-R at day 29: 19.5 (13.1, 25.9)	No discontinuations due to AEs Two patients discontinued the study drug No discontinuations due to AEs
Donaldson, 2018 [27]	2	*CFTR*: F/F Age: 18+ Sample size: 131 *CFTR*: F/F Age: 18+ Sample size: 67 *CFTR*: F/G551D Age: 12+ Sample size: 18	*Dose escalation:* TEZ 10 mg SID TEZ 30 mg SID TEZ 100 mg SID TEZ 150 mg SID TEZ 10 mg SID/IVA 150 mg BID TEZ 30 mg SID/IVA 150 mg BID TEZ 100 mg SID/IVA 150 mg BID TEZ 150 mg SID/IVA 150 mg BID Placebo *Dosage regimen testing:* TEZ 100 mg SID/IVA 150 mg BID TEZ 10 mg SID/IVA 50 mg BID TEZ 50 mg SID/IVA 150 mg BID Placebo TEZ 100 mg SID/IVA 150 mg BID Placebo/IVA 150 mg BID	28 days of treatment 28 days of washout 28 days of treatment 28 days of washout 28 days of treatment 28 days of washout	(1) safety through day 56: not significant between the groups (1) change in ST through day 28: the highest reduction was reported in TEZ 100 mg: −19.58 (−24.57, −14.59) (2) absolute change in ppFEV1 through day 28: the highest improvement was reported in TEZ 100 mg/IVA 150 mg: 3.89 (0.94, 6.83) (2) relative change in ppFEV1 through day 28: the highest improvement was reported in TEZ 100 mg/IVA 150 mg: 7.04 (1.77, 12.31) (1) safety through day 56: not significant between what was reported in TEZ 50 mg/IVA 150 mg: 1.02 (−6.04, 8.08) (1) safety through day 56: not significant between the groups. (1) change in ST through day 28: -17.2 (−31.75, −2.65) (2) absolute change in ppFEV1 through day 28: 3.2 (−4.1, 10.51) (2) relative change in ppFEV1 through day 28: 3.72 (−7.77, 15.21)	Four patients discontinued the study drug One patient discontinued the study drug No discontinuations due to AEs
Keating, 2018 [12]	2	*CFTR*: F/MF Age: 18+ Sample size: 95 *CFTR*: F/F Age: 18+ Sample size: 28	*F/MF* ELX 50 mg SID/TEZ 100 mg SID/IVA 150 mg BID ELX 100 mg SID/TEZ 100 mg SID/IVA 150 mg BID ELX 200 mg SID/TEZ 100 mg SID/IVA 150 mg BID ELX 200 mg SID/TEZ 100 mg SID/VX-561 * SID Placebo *F/F* ELX 200 mg SID/TEZ 100 mg SID/IVA 150 mg BID TEZ 100 mg SID/IVA 150 mg BID	4 weeks	*F/MF* (1) absolute change in ppFEV1: 13.8 (10.9, 16.6) (2) ST: −39.1 mmol/L (−44.9, −33.3) (2) 25.7 points (18.3, 33.1) *FF* (1) absolute change in ppFEV1: 11.0 (7.9, 14.0) (2) ST: −39.6 mmol/L (−45.3, −33.8) (2) CFQ-R: 20.7 points (12.5, 29.0)	Three patients discontinued the study drug (rash in one patient, elevated bilirubin level in none patient, and chest pain in one patient)
Walker, 2019 [28]	3	*CFTR*: F/MF + F/F Age: 6–11 Sample size: 13 (part A) + 70 (part B)	TEZ 50 mg SID/IVA 75 mg BID TEZ 75 mg SID/IVA 150 mg BID	24 weeks	(1) safety: no difference in AEs between groups in part A and B (2) absolute change ppFEV1: 0.9 (−0.6, 2.3) (2) relative change ppFEV1: 1.4 (−0.4, 3.1) (2) increase in BMI: 0.23 (0.06, 0.40) (2) ST: -14.5 (−17.4, −11.6) (2) CFQ-R: 3.4 (1.4, 5.5)	No discontinuations due to AEs
Middleton, 2019 [13]	3	*CFTR*: F/MF Age: 12+ Sample size: 403	ELX 200 mg SID/TEZ 100 mg SID/IVA 150 mg BID Placebo	24 weeks	(1) absolute change in ppFEV1 at week 4: 13.8 (12.1, 15.4) (2) absolute change in ppFEV1 through week 24: 14.3 (12.7, 15.8) (2) decrease in PEx through week 24: 0.37 (0.25, 0.55) (2) absolute change in ST through week 24: −41.8 (−44.4, −39.3) (2) absolute change in CFQ-R through week 24: 20.2 (17.5, 23) (2) absolute change in BMI at week 24: 1.04 (0.85, 1.23) (2) absolute change in ST at week 4: −41.2 (−44, −38.5) (2) absolute change in CFQ-R at week 4: 20.1 (16.9, 23.2) (2) time to first PEx through week 24: data not shown (2) absolute change in BMI-for-age z score at week 24: data not shown (2) absolute change in body weight at week 24: data not shown	Two patients discontinued the study drug for rash in one patient, portal hypertension in one patient (0.5%)
Heijerman, 2019 [15]	3	*CFTR*: F/F Age: 12+ Sample size: 107	ELX 200 mg SID/TEZ 100 mg SID/IVA 150 mg BID TEZ 100 mg SID/IVA 150 mg BID	4 weeks	(1) absolute change in ppFEV1 at week 4: 10.4 (8.6, 12.2) (2) absolute change in ST at week 4: −43.4 (−46.9, −40.0) (2) absolute change in CFQ-R through week 4: 16.0 (12.1, 19.9)	No discontinuations due to AEs
Bell (I), 2019 [29]	2a	*CFTR*: F/F Age: 18+ Sample size: 59	GLPG2222 50/100/200/400 mg BID Placebo	4 weeks	(1) safety (2) decrease in ST: −15.8 mmol/L (−23.2, −8.3) (2) change in ppFEV1: NS (2) CFQ-R: NS	No discontinuations due to AEs
Bell (II), 2019 [29]	2a	*CFTR*: F/gating mutation on IVA Age: 18+ Sample size: 27	GLPG2222 150/300 mg BID Placebo	4 weeks	(1) safety (2) ST: -11.7 mmol/L (−21.1, −2.2) (2) change in ppFEV1: NS (2) CFQ-R: NS	No discontinuations due to AEs
Davies, 2019 [30]	2a	*CFTR*: at least one G551D Age: 18+ Sample size: 26	*One-week IVA washout* GLPG1837 increasing dose Single-arm	4 weeks	(1) safety (2) ST: −28.7 mmol/L (−39.1, −18.4) (2) absolute change in ppFEV1: 4.6 (0.2, 5.3)	One patient interrupted the study drug (non-cardiac increase in CPK)
van Konigsbruggen-Rietschel, 2019 [31]	2a	*CFTR*: F/F on LUM/IVA Age: 18+ Sample size: 22	GLPG2737 25 mg BID + LUM 400 mg BID/IVA 150 mg BID LUM 400 mg BID/IVA 150 mg BID	4 weeks	(1) ST: −19.0 mmol/L (−36, −3.2) (2) absolute change in ppFEV1: 3.4 (−0.5, 7.3) CFQ-R: −1.1 (−2.1, 18.9)	No discontinuations due to AEs

Definitions: F = F508del *CFTR* mutation; RF = residual function mutation according to the trial list [10]; MF = minimal function *CFTR* mutation according to the trial list [12]; IVA = ivacaftor; LUM = lumacaftor; TEZ = tezacaftor; ELX = elaxacaftor; PEX = pulmonary exacerbation; ST = sweat test; NDP: nasal potential difference; CFQ-RD = Cystic Fibrosis Questionnaire Revised; SID = once daily; BID = twice daily. * a deuterated form of the *CFTR* potentiator ivacaftor.

**Table 2:** Cumulative incidence of 15 most common adverse events among the four *CFTR* modulators currently available in clinical practice.

Event	IVA (*n* = 483)	LUM-IVA (*n* = 1111)	TEZ-IVA (*n* = 607)	ELX-TEZ-IVA (*n* = 276)
Number of Patients (Percent)				
PEX	104 (21.5%)	355 (31.9%)	137 (22.6%)	59 (21.4%)
Cough	102 (21.1%)	224 (20.2%)	134 (22.1%)	57 (20.6%)
Oropharyngeal pain	46 (9.5%)	94 (8.5%)	31 (5.1%)	20 (7.2%)
Increased sputum	44 (9.1%)	166 (14.9%)	62 (10.2%)	60 (21.7%)
Nasal congestion	43 (8.9%)	82 (7.4%)	16 (2.6%)	0
Headache	44 (9.1%)	140 (12.6%)	79 (13%)	35 (12.7%)
Upper respiratory tract infection	43 (8.9%)	78 (7%)	0	24 (8.7%)
Hemoptysis	26 (5.7%)	121 (10.9%)	38 (6.3%)	11 (4%)
Dyspnea	4 (0.8%)	126 (11.3%)	9 (1.5%)	0
Chest tightness	0	104 (9.4%)	0	0
Abdominal pain	34 (7%)	28 (2.5%)	10 (1.6%)	0
Fatigue	34 (7%)	9 (0.8%)	35 (5.8%)	9 (3.2%)
Pyrexia	34 (7%)	30 (2.7%)	50 (8.2%)	9 (3.2%)
Diarrhea	29 (6%)	99 (8.9%)	19 (3.1%)	26 (9.4%)
Increased AST or ALT > 3 ULN or increased bilirubin > 1.5 ULN	39 (8.1%)	81 (7.3%)	29 (4.8%)	54 (19.6%)

Definitions: PEX: pulmonary exacerbations; ULN: upper level of normality.
